# Tuberculin Skin Testing and Treatment Modulates Interferon-Gamma Release Assay Results for Latent Tuberculosis in Migrants

**DOI:** 10.1371/journal.pone.0097366

**Published:** 2014-05-09

**Authors:** Matthew K. O'Shea, Thomas E. Fletcher, Nicholas J. Beeching, Martin Dedicoat, David Spence, Helen McShane, Adam F. Cunningham, Duncan Wilson

**Affiliations:** 1 The Jenner Institute, Nuffield Department of Medicine, University of Oxford, Oxford, United Kingdom; 2 School of Immunity and Infection, MRC Centre for Immune Regulation, University of Birmingham Edgbaston, Birmingham, United Kingdom; 3 Liverpool School of Tropical Medicine, Liverpool, United Kingdom; 4 Department of Infectious Disease and Tropical Medicine, Heartlands Hospital, Birmingham, United Kingdom; 5 Department of Respiratory Medicine, The Friarage Hospital, Northallerton, United Kingdom; 6 Royal Centre for Defence Medicine (Academia and Research), Joint Medical Command, Birmingham, United Kingdom; Maastricht University Medical Center (MUMC), Netherlands

## Abstract

**Background:**

Identifying latent tuberculosis infection (LTBI) in people migrating from TB endemic regions to low incidence countries is an important control measure. However, no prospective longitudinal comparisons between diagnostic tests used in such migrant populations are available.

**Objectives:**

To compare commercial interferon (IFN)-gamma release assays (IGRAs) and the tuberculin skin test (TST) for diagnosing LTBI in a migrant population, and the influence of antecedent TST and LTBI treatment on IGRA performance.

**Materials and Methods:**

This cohort study, performed from February to September 2012, assessed longitudinal IGRA and TST responses in Nepalese military recruits recently arrived in the UK. Concomitant T-SPOT.TB, QFT-GIT and TST were performed on day 0, with IGRAs repeated 7 and 200 days later, following treatment for LTBI if necessary.

**Results:**

166 Nepalese recruits were prospectively assessed. At entry, 21 individuals were positive by T-SPOT.TB and 8 individuals by QFT-GIT. There was substantial agreement between TST and T-SPOT.TB positives at baseline (71.4% agreement; κ = 0.62; 95% CI:0.44–0.79), but only moderate concordance between positive IGRAs (38.1% agreement; κ = 0.46; 95% CI:0.25–0.67). When reassessed 7 days following TST, numbers of IGRA-positive individuals changed from 8 to 23 for QFT-GIT (p = 0.0074) and from 21 to 23 for T-SPOT.TB (p = 0.87). This resulted in an increase in IGRA concordance to substantial (64.3% agreement; κ = 0.73; 95% CI:0.58-0.88). Thus, in total on day 0 and day 7 after testing, 29 out of 166 participants (17.5%) provided a positive IGRA and of these 13 were TST negative. Two hundred days after the study commenced and three months after treatment for LTBI was completed by those who were given chemoprophylaxis, 23 and 21 participants were positive by T-SPOT.TB or QFT-GIT respectively. When individual responses were examined longitudinally within this population 35% of the day 7 QFT-GIT-positive, and 19% T-SPOT.TB-positive individuals, were negative by IGRA. When the change in the levels of secreted IFN-γ was examined after chemoprophylaxis the median levels were found to have fallen dramatically by 77.3% from a pre-treatment median concentration of IFN-γ 2.73 IU/ml to a post-treatment median concentration IFN-γ 0.62 (p = 0.0002).

**Conclusions:**

This study suggests differences in the capacity of commercially available IGRAs to identify LTBI in the absence of antecedent TST and that IGRAs, in the time periods examined, may not be the optimal tests to determine the success of chemoprophylaxis for LTBI.

## Introduction

The effective control and elimination of tuberculosis (TB) in low-incidence countries requires the prompt identification of individuals with active and latent TB infection (LTBI). This latter group has the potential to reactivate their infection, progress to disease and transmit the organism to others. In the United Kingdom (UK), TB notifications have increased to a plateau of 9,000 new cases per year of which >70% now occur in foreign-born individuals, in whom the incidence rate of TB is >20 times higher than among those born in the UK [1]. Therefore, effective assessment of LTBI in migrant populations is of significant health and social importance.

Nonetheless, the diagnosis of LTBI remains challenging. Until recently, the tuberculin skin test (TST) was the diagnostic method of choice for LTBI. Although cheap and easy to perform, TST has limited sensitivity and specificity and is not suitable for repeat testing [2], [3]. Recently, interferon-gamma (IFN-γ) production from T cells in response to the *Mycobacterium tuberculosis (M.tb)*-specific early secretory antigenic target-6 (ESAT-6), culture filtrate protein-10 (CFP-10) and TB7.7 antigens has been employed in IFN-γ release assays (IGRAs). IGRAs have some advantages over TST including the speed in which results are obtained, theoretical independence from prior Bacillus Calmette-Guérin (BCG) vaccine exposure, and their potential for intra-patient repeat use [4]–[8]. Nevertheless, some issues remain for their use in diagnosing LTBI, primarily relating to subject variability, reproducibility, and the potential discordance between assays [9]–[14].

There are multiple strategies for diagnosing LTBI. Some countries recommend a single-step IGRA-based LTBI screening strategy, whilst others advocate a primary TST and subsequent IGRA in TST-positive individuals, which is the current UK recommendation [15]–[18]. Limited data exist for direct comparison between TST and commercially available IGRAs for diagnosing LTBI in migrant populations. Available data have indicated that lower detection rates are found using QFT-GIT than T-SPOT.TB, but that both assays are potentially less sensitive than TST [19]. Moreover, it remains unclear what the impact of antecedent TST, which includes antigens used in IGRAs, is on subsequent IGRA results and this important question remains to be fully elucidated [10], [20]–[27].

Once LTBI has been diagnosed and treated, clear correlates that identify whether treatment has been successful, or not, are desirable yet sadly lacking [28]. Data suggest that following successful treatment of active disease, mycobacterial antigen load declines and with it the frequency of effector T cells [29]–[33]. This indicates that the quantification of T cell responses to *M.tb*-specific antigens may also provide some correlative measure of bacterial burden and so help determine the effectiveness of anti-tuberculosis treatment [30]. Nevertheless, changes in IGRA measurements do not correlate with classical sputum markers of bacillary burden in active disease and do not necessarily change after treatment for active disease or LTBI [34]–[37]. More data are needed to determine whether altered T cell responses are relevant.

For almost two centuries the British Army has recruited young Nepalese males into the Gurkha regiment. Nepal is a region of high TB endemicity with an incidence of 163 cases per 100,000 population per year and before leaving Nepal for the UK, recruits are examined to exclude active TB disease. In the UK they are further assessed for LTBI. This provides a unique opportunity to examine IGRA responses longitudinally, with and without chemoprophylaxis, in a well-defined cohort of individuals, whose environmental experiences are controlled in a manner not normally possible in civilian populations. Additionally, all individuals can be sampled in parallel on the same day. This synchronous processing enables direct comparisons to be made between IGRAs and TST in the same patient cohort and whether chemoprophylaxis influences subsequent IGRAs and offers an opportunity to optimally diagnose LTBI in populations of migrants leaving countries of relatively high TB incidence to a country of low TB incidence. The objectives of the current study were to examine the longitudinal IGRA responses in Gurkha recruits after recent entry into the UK and the effect of TST and chemoprophylaxis on the results obtained.

## Materials and Methods

### Ethics, study design & population

**Figure 1:**
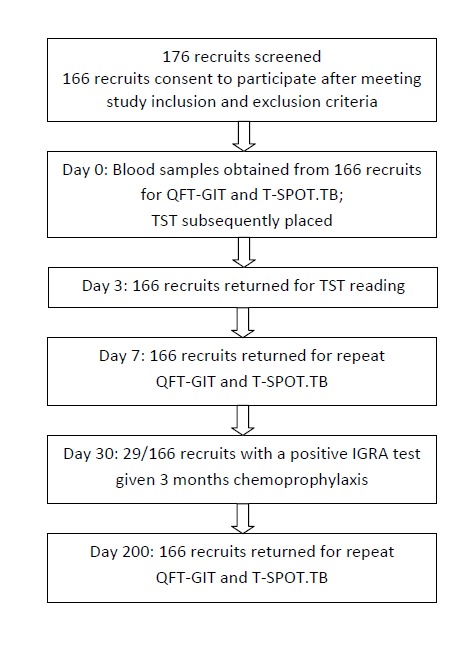
Study flow diagram of screening and testing for latent tuberculosis infection. At recruitment demographic data, past medical history, previous TB disease, BCG vaccination status and recent TB exposure were all recorded. TST  =  tuberculin skin test; QFT-GIT  =  QuantiFERON Gold in-Tube.

Ethical approval was granted by the Ministry of Defence Research Ethics Committee (MODREC 237/PPE/11). This was a prospective, longitudinal cohort study among Nepalese military recruits who had left Nepal and recently entered the UK. Inclusion criteria included age at enrolment of ≥18 years and exclusion of active TB disease, and in accordance with current military and UK migrant screening policy, HIV status was not assessed. The estimated prevalence of HIV infection among adults aged 15–49 years in Nepal is 0.3% [38]. The Nepalese vaccination policy for TB is to immunize once at birth with BCG. The full study schedule is shown in Figure 1. Of the 176 Gurkha recruits arriving in the UK in 2012, 166 fulfilled the inclusion criteria, agreed to participate and provided written informed consent.

### IGRA testing of recruits in the UK

Two commercially available assays were used - The QuantiFERON-TB Gold In-Tube (QFT-GIT; Cellestis, Carnegie, Australia) whole blood enzyme-linked immunosorbent assay (ELISA), and the T-SPOT.TB assay (Oxford Immunotec, Abingdon, UK) enzyme-linked immunospot (ELISPOT) assay using peripheral blood mononuclear cells (PBMCs). Peripheral venous blood samples were provided at the first visit (day 0), and on days 7 and 200. Samples were transported to Oxford Immunotec's diagnostic laboratory for the T-SPOT.TB assay within 24 hours and the Health Protection Agency regional mycobacterial reference laboratory (Newcastle, UK) for the QFT-GIT assay within 8 hours of collection.

Standard criteria for positive responses were used for both tests as recommended by the manufacturers: for QFT-GIT, IFN-γ concentrations ≥0.35 IU/ml and ≥25% of the negative control value, after correcting for negative control values; for T-SPOT.TB, ≥6 SFCs/250,000 PBMCs to ESAT-6 or CFP-10, adjusted for the negative control, except when the negative control was ≥6 SFCs, whereupon SFCs of ≥ twice the negative control were required. Between serial IGRAs, conversion to positivity was defined as an initial IFN-γ concentration of <0.35 IU/ml or <6 SFCs which increased to ≥0.35 IU/ml or ≥6 SFCs, respectively. The opposite definition was used for reversion to negativity. ‘Borderline’ or ‘uncertainty’ zones have previously been defined as 0.2–0.7 IU/ml for QFT-GIT or 4–8 SFCs for T-SPOT.TB. Conversion thresholds of an increase of <0.2 to >0.7 IU/ml for QFT-GIT and from <6 to >9 SFCs for T-SPOT.TB, were also applied in our analyses [11], [25].

### Tuberculin skin testing

On day 0, following blood sampling for the IGRAs, a TST was performed by the Mantoux method. Two tuberculin units of strain RT23 purified protein derivative (PPD, Statens Serum Institut, Copenhagen, Denmark) was applied intradermally and transverse induration measured 72 hours later. Positivity was defined as an induration of ≥6 mm in BCG-naive and ≥15 mm in BCG-vaccinated individuals in accordance with UK guidelines (stratified threshold). We also used non-stratified threshold for TST positivity, defined as induration ≥10 mm irrespective of BCG vaccination history.

### Treatment of LTBI

Individual's positive for either IGRA at day 0 and/or day 7, with normal chest radiography and no indication of active disease, were defined as having LTBI and offered LTBI treatment. Individuals who only provided a positive TST, but not a positive IGRA, were not treated. A three month regimen of daily self-administered weight-based, fixed-dose combination therapy of rifampicin and isoniazid was prescribed in accordance with current UK guidance [17].

### Statistical Analysis

Data analyses were performed using GraphPad Prism. The two-tailed Fisher's exact test was used to compare categorical data and the Mann-Whitney test (unpaired) or Wilcoxon signed rank test (matched-pairs) for analysis. McNemar's test was used to compare paired proportions. Concordance between TST and IGRA results was assessed using percentage agreement and Cohen's κ coefficients; strength of agreement was considered ‘poor’ for *κ*≤0.20, ‘fair’ for 0.20<*κ*≤0.40, ‘moderate’ for 0.40<*κ*≤0.60, ‘substantial’ for 0.60<*κ*≤0.80 and ‘optimal’ for 0.80<*κ*≤1.00 [39].

## Results

Of 176 Gurkha recruits who arrived in the UK from Nepal, 166 volunteered to participate in the study. All participants were male, aged 18–21 years and 105 had been vaccinated with BCG. No participant reported a prior history of TB, known exposure to TB or any other past medical history. All individuals enrolled at day 0 re-attended at days 3, 7 and 200 and provided a complete set of samples (Figure 1). In each case the samples for each time-point were obtained on the same day.

### Differences in QFT-GIT and T-SPOT.TB tests to detect anti-mycobacterial responses

The IGRA testing revealed that 21 individuals were T-SPOT.TB positive (12.9%, two ‘borderline positive’) and eight were QFT-GIT positive (4.8%). All QFT-GIT positives were also T-SPOT.TB positive. Thus, 61.9% T-SPOT.TB positive participants were QFT-GIT negative, meaning the tests showed moderate agreement (90.4%; κ = 0.46; 95% CI:0.25–0.67) (Table 1).

**Table 1:** Concordance between TST, T-SPOT.TB and QFT-GIT results stratified by study visit and BCG vaccination status.

Time-point	Assay (n = 166)	Agreement					
		TST		T-SPOT.TB (Day 0)		QFT-GIT (Day 0)	
		%	κ (95% CI)	%	κ (95% CI)	%	κ (95% CI)
**Day 0 Baseline**	T-SPOT.TB (all)	91.0	0.62 (0.44–0.79)				
	T-SPOT.TB (BCG-naive)	90.2	0.73 (0.53–0.93)				
	T-SPOT.TB (BCG-vaccinated)	91.4	0.44 (0.14–0.73)				
	QFT-GIT (all)	89.8	0.37 (0.14–0.60)	90.4	0.46 (0.25–0.67)		
	QFT-GIT (BCG-naive)	82.0	0.40 (0.15–0.66)				
	QFT-GIT (BCG-vaccinated)	94.3	0.22 (−0.18–0.63)				
**Day 7 Post-TST**	T-SPOT.TB (all)	91.0	0.62 (0.44–0.79)	91.6	0.66 (0.50–0.83)	93.4	0.73 (0.58–0.88)
	T-SPOT.TB (BCG-naive)	91.8	0.78 (0.60–0.96)				
	T-SPOT.TB (BCG-vaccinated)	90.5	0.33 (0.02–0.64)				
	QFT-GIT (all)	91.6	0.63 (0.46–0.81)	93.4	0.73 (0.58–0.88)	91.0	0.48 (0.26–0.70)
	QFT-GIT (BCG-naive)	91.8	0.78 (0.59–0.96)				
	QFT-GIT (BCG-vaccinated)	91.4	0.36 (0.04–0.68)				
**Day 200**	T-SPOT.TB (all)	89.8	0.57 (0.38–0.75)	91.6	0.66 (0.50–0.83)	88.6	0.51 (0.32–0.70)
	T-SPOT.TB (BCG-naive)	90.2	0.73 (0.53–0.93)				
	T-SPOT.TB (BCG-vaccinated)	89.5	0.31 (0.01–0.60)				
	QFT-GIT (all)	84.9	0.30 (0.10–0.51)	88.6	0.51 (0.32–0.70)	88.6	0.30 (0.07–0.52)
	QFT-GIT (BCG-naive)	85.3	0.53 (0.29–0.78)				
	QFT-GIT (BCG-vaccinated)	84.8	0.05 (−0.16–0.25)				

TST  =  tuberculin skin test; QFT-GIT  =  QuantiFERON Gold in-Tube; BCG  =  Bacillus Calmette-Guérin.

**Figure 2:**
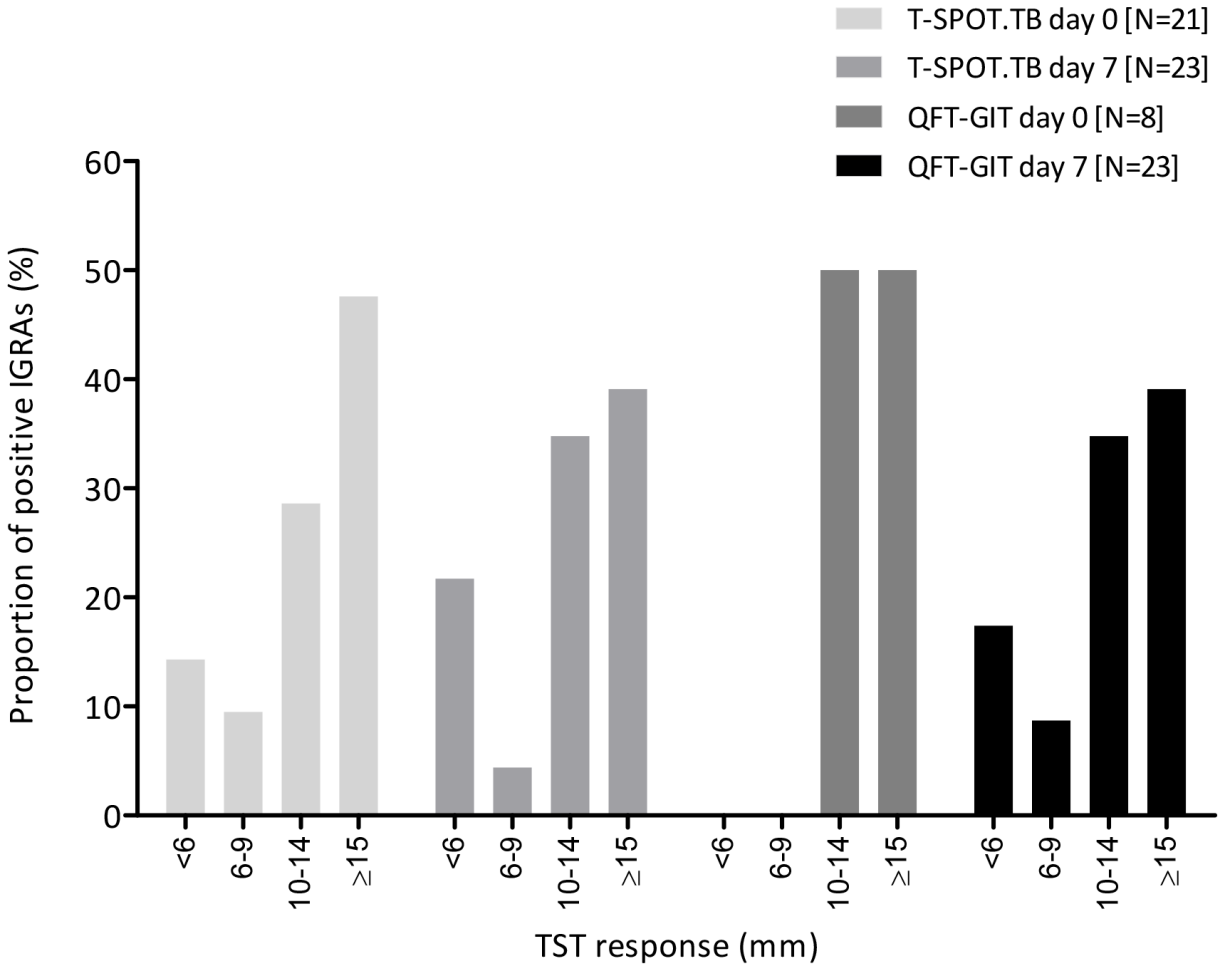
The relationship between IGRA response and TST induration. The distribution of positive IGRAs (pre- and post-TST) by size of TST induration. TST =  tuberculin skin test; QFT-GIT =  QuantiFERON Gold in-Tube.

**Figure 3:**
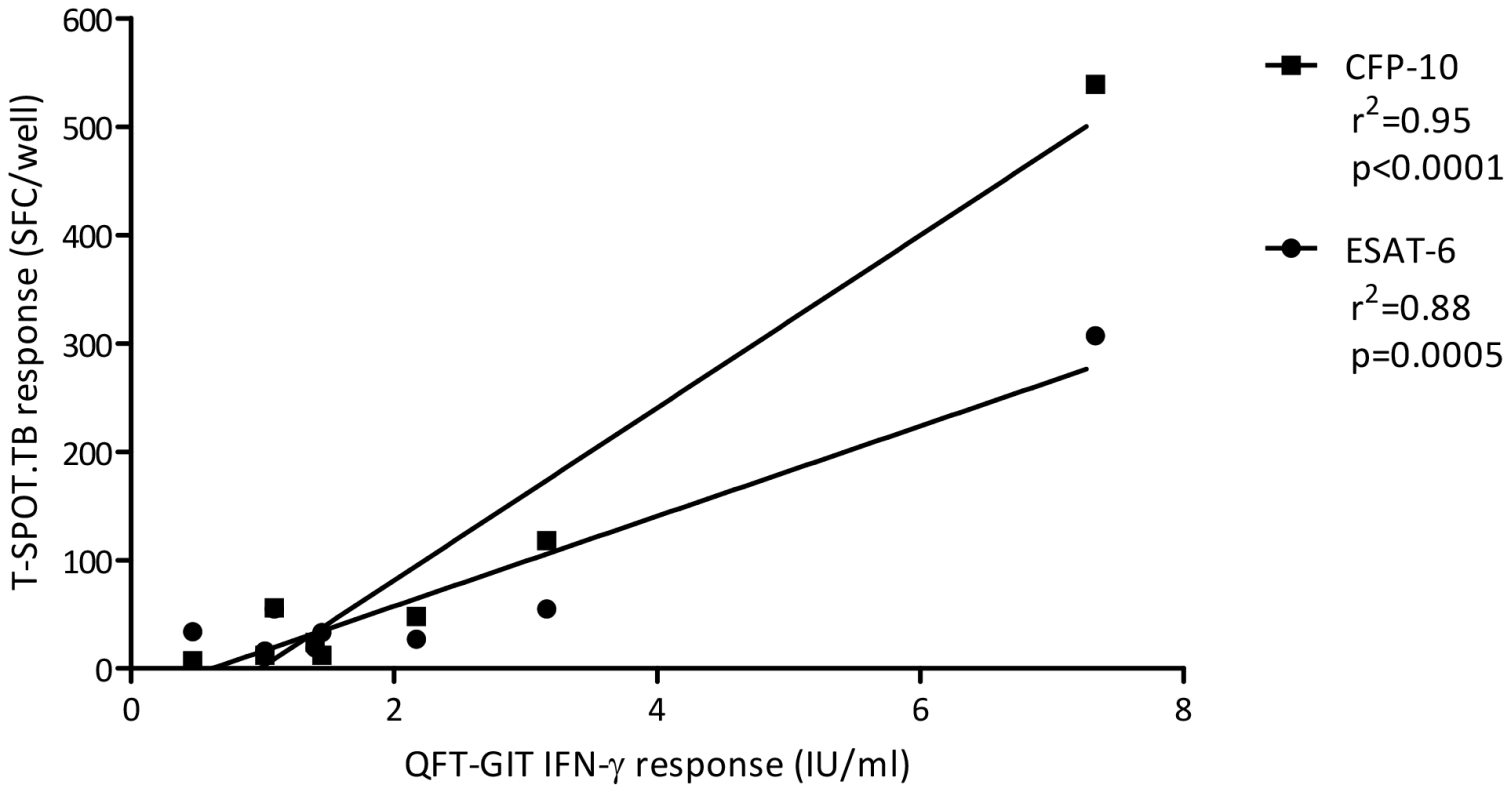
The relationship between the magnitude of IFN-γ response among individuals positive by both IGRAs at baseline. Solid line represents linear correlation. QFT-GIT =  QuantiFERON Gold in-Tube; IFN-γ =  interferon-gamma; ESAT-6 =  early secretory antigenic target-6; CFP-10 =  culture filtrate protein-10; IU =  international units; SFC =  spot forming cells.

TST were performed at day 0 and read at day 3, and the results compared to the day 0 IGRAs. There was substantial agreement between TST and T-SPOT.TB and fair agreement between TST and QFT-GIT (Table 1). Twenty-one (12.9%) individuals were TST positive using a stratified threshold which increased to 26 (15.9%) with a non-stratified threshold, of whom 5 and 13 had been BCG vaccinated (respectively). IGRA-positivity occurred at all sizes of TST induration (Figure 2), whereas IGRA-negative TST-positive individuals tended to have a lower induration. Agreement between both IGRAs and TST was highest in BCG-naive individuals and greater for T-SPOT.TB than for QFT-GIT (Table 1). Therefore, T-SPOT.TB had a greater concordance with TST than did QFT-GIT when performed prior to the TST. There was a strong positive linear correlation between IFN-γ and ESAT-6 and CFP-10 responses among individuals positive by both IGRAs at baseline (r^2^ = 0.88 and 0.95, respectively; Figure 3).

### Antecedent TST augments IGRA concordance

To examine if the TST influenced the results of a subsequent IGRA, repeat IGRAs were performed 7 days after TST administration. Four (19.0%) of the 21 T-SPOT.TB positive individuals at day 0 were no longer positive on repeat IGRA, two of whom had been ‘borderline positive’ on day 0. Six (4.2%) of the 142 IGRA-negative individuals on day 0 became positive by T-SPOT.TB, including one ‘borderline positive’ result, yielding a total of 23 positives at day 7 (13.9%). One of six converters was TST positive.

None of the eight day 0 positive QFT-GIT individuals reverted to negativity. However 15 (9.5%) previously negative individuals converted to positivity, nearly tripling the numbers of positive individuals to 23 (13.9%) on day 7 (p = 0.0074). Of the 23 QFT-GIT-positive individuals, nine were also TST-positive (60.0% concordance). Three of six T-SPOT.TB converters also provided positive QFT-GIT tests, thus increasing the agreement between the IGRA tests and TST to substantial (κ = 0.62-0.63; 95% CI:0.44–0.81) (Table 1). Concordance between T-SPOT.TB and TST were similar at day 0 and 7, but the strength of agreement between QFT-GIT and TST improved considerably at day 7 (day 0, κ = 0.37; 95% CI:0.14–0.60; day 7, κ = 0.63; 95% CI:0.46–0.81). Agreement between QFT-GIT and T-SPOT.TB results at day 7 also improved to 93.4% (κ = 0.73; 95% CI:0.58–0.88). Combining the day 7 IGRA results gave a total of 29 positives among the study population (17.5%). Concordant pairs (positive by both IGRAs at day 7) occurred in 18 cases. Overall, intra-assay agreement between day 0 and day 7 T-SPOT.TB results was higher than for QFT-GIT results (κ = 0.66; 95% CI:0.50–0.83; κ = 0.48; 95% CI:0.26–0.70, respectively) (Table 1). Therefore, an antecedent TST was associated with a profound increase in QFT-GIT IGRA detections.

### Recent tuberculin exposure is associated with augmented IGRA responses

**Figure 4:**
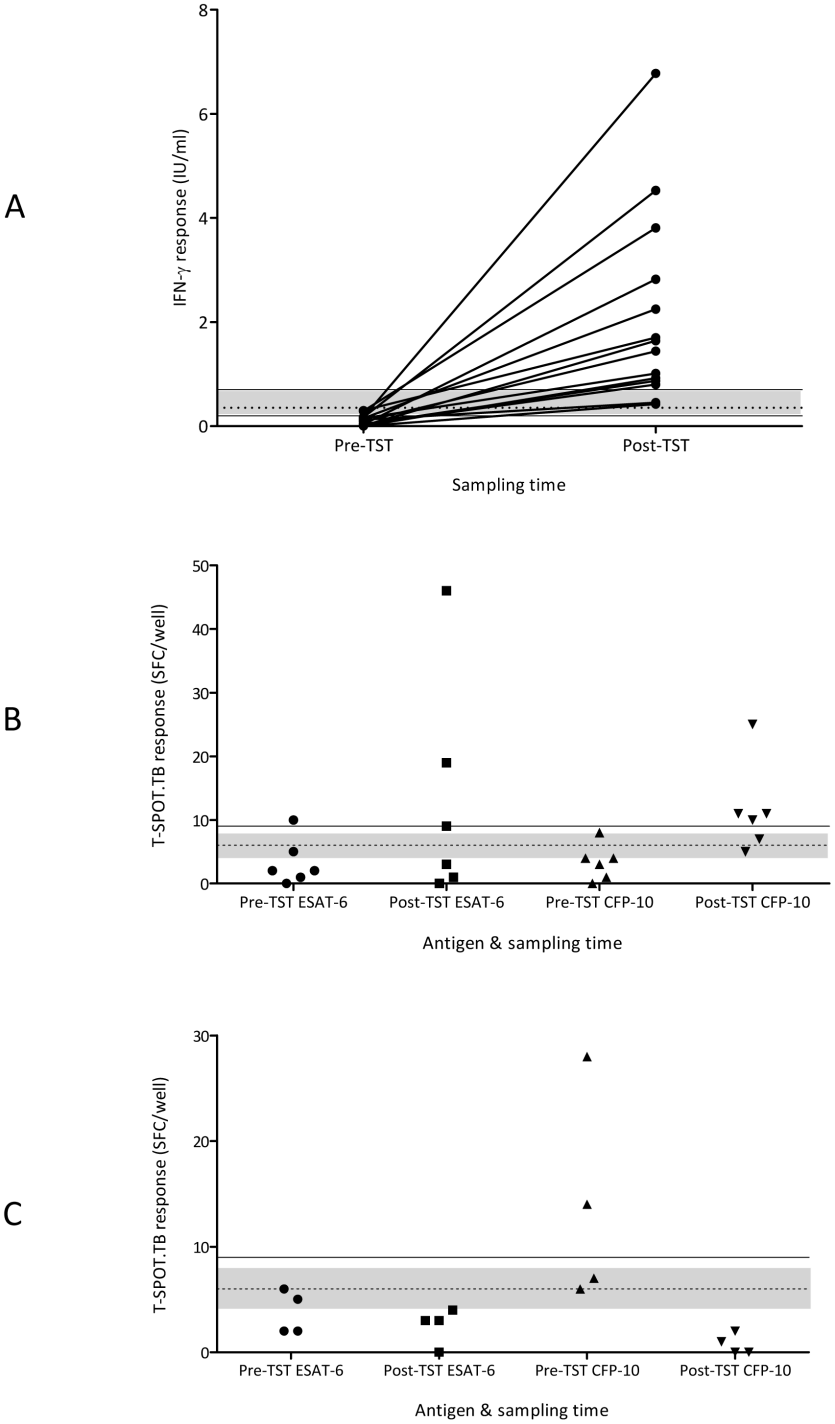
IGRA responses before and after TST administration. (A) IFN-γ responses detected by QFT-GIT in individuals who became positive following TST; (B) SFC numbers in response to ESAT-6 and CFP-10 in individuals who became positive by T-SPOT.TB following TST; (C) SFC numbers in response to ESAT-6 and CFP-10 in individuals who became negative by T-SPOT.TB following TST. Uncertainty zone (grey shaded area); threshold for QFT-GIT positivity (dashed line); upper and lower thresholds for T-SPOT.TB conversion (solid lines). TST =  tuberculin skin test; QFT-GIT =  QuantiFERON Gold in-Tube; IFN-γ =  interferon-gamma; ESAT-6 =  early secretory antigenic target-6; CFP-10 =  culture filtrate protein-10; IU =  international units; SFC =  spot forming cells.

Since antecedent TST could alter IGRA positivity we also assessed whether the strength of the IGRA response was also affected. Changes in positive results were observed for both IGRA tests and were often, though not always, associated with increased numbers of results falling into the zones of uncertainty (Table 2). The eight individuals positive by QFT-GIT at both day 0 and day 7 had enhanced IFN-γ levels after the TST (day 0 median IFN-γ 1.43 IU/ml, day 7 median IFN-γ 7.67 IU/ml, p = 0.008). Of the 15 participants initially negative at day 0 by QFT-GIT but subsequently positive after TST, there was a>15 fold increase in IFN-γ levels (Figure 4A; day 0 median IFN-γ 0.09 IU/ml, day 7 median IFN-γ 1.44 IU/ml, p = <0.0001). In contrast, in the 143 individuals QFT-GIT who remained negative at day 7 no change in median IFN-γ levels was observed (−0.01 IU/ml both day 0 and 7, p = 0.85).

**Table 2:** Frequency of IGRA conversions and reversions following TST administration and stratified by baseline QFT-GIT and T-SPOT.TB responses.

Baseline IGRA response	n	TST positive n (%)	IGRA positive	Conversion frequency n (%)	Reversion frequency n (%)
**QFT-GIT** IFN-γ (IU/ml)					
<0.01	112	5 (4.5)		3 (2.7)	na
0.01–0.09	38	5 (13.2)		5 (13.2)	na
0.1–0.19	6	3 (50.0)		5 (83.3)	na
0.2–0.34	2	2 (100.0)		2 (100.0)	na
0.35–0.69	1	1 (100.0)	1	na	0
0.7–0.9	0	0	0	na	0
1.0–2.9	5	3 (60.0)	5	na	0
3.0–5.9	1	1 (100.0)	1	na	0
6.0–10.0	1	1 (100.0)	1	na	0
**Total**	**166**	**21**	**8**	**15**	**0**
**T-SPOT.TB**					
ESAT-6 (SFC/well)					
0–3	141	10 (7.1)		2 (1.4)	na
4–5	7	2 (28.6)		1 (14.3)	na
6–8	3	0 (0.0)	1	na	1 (100)
9–29	6	4 (66.7)	4	na	0
≥30	6	5 (83.3)	7	na	0
**Total**	**163**	**21**	**12**	**3**	**1**
CFP-10 (SFC/well)					
0–3	130	6 (4.6)		2 (1.5)	na
4–5	7	0 (0.0)		2 (28.6)	na
6–8	8	3 (37.5)	5	1 (20.0)	2 (40.0)
9–29	10	5 (50.0)	8	na	2 (25.0)
≥30	8	7 (87.5)	8	na	0
**Total**	**163**	**21**	**21**	**5**	**4**

IGRA =  interferon-gamma release assay; TST =  tuberculin skin test; QFT-GIT =  QuantiFERON Gold in-Tube; IFN-γ =  interferon-gamma; ESAT-6 =  early secretory antigenic target-6; CFP-10 =  culture filtrate protein-10; IU =  international units; SFC =  spot forming cells; na =  not applicable. Note: n = 163 for T-SPOT.TB tests due to the exclusion of three indeterminate results.

Among the six individuals converting to T-SPOT.TB positivity at day 7, numbers of SFCs increased for both ESAT-6 and CFP-10, with medians rising by up to 3-fold (ESAT-6 Day 0 = 2 SFCs/well, day 7 = 6 SFCs/well; CFP-10 Day 0 = 3.5 SFCs/well, Day 7 = 10.5 SFCs/well, p = 0.03). Among the 17 T-SPOT.TB-positives at both day 0 and 7, median SFCs for each antigen over the two sampling points remained relatively stable (ESAT-6 Day 0 = 19 SFCs/well, Day 7 = 21 SFCs/well; CFP-10 Day 0 = 26 SFCs/well, Day 7 = 32 SFCs/well). However, among the eight individuals positive by both IGRAs at days 0 and 7 there was augmentation in SFC numbers for both antigens, but particularly to ESAT-6 (median SFCs/well to ESAT-6, day 0 = 34, day 7 = 145, p = 0.31; median SFCs/well to CFP-10, day 0 = 36, day 7 = 42, p = 0.38). There was no significant change in SFC numbers in the 134 persistently negative individuals.

A significant proportion of the dynamic IGRA responses (conversions and reversions) following TST occurred around the zones of uncertainty for both assays (Table 2). However, even with the application of very stringent definitions for conversion thresholds, ‘true’ conversions did occur and more frequently with the QFT-GIT than T-SPOT.TB assay (Figure 4B and C).

### Treatment for LTBI does not alter a positive response in most individuals

**Figure 5:**
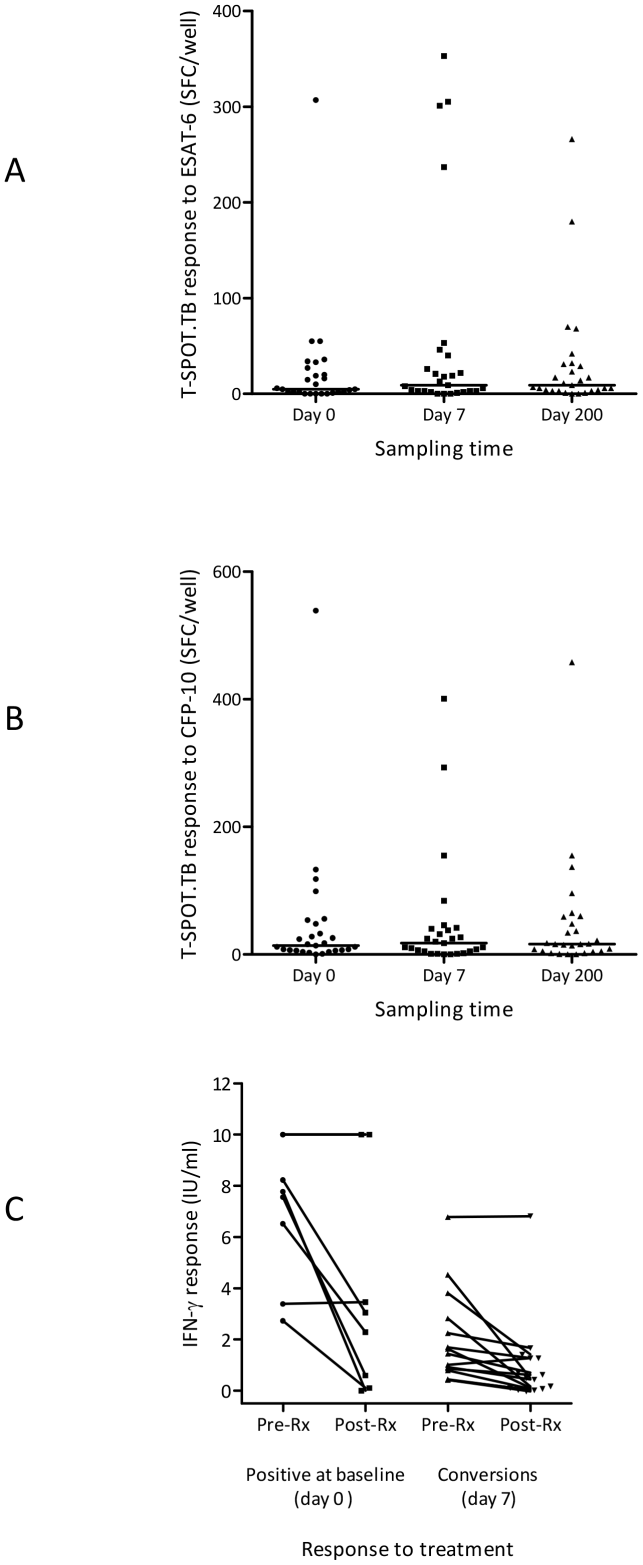
IGRA responses following LTBI treatment in individuals positive by IGRA at any stage of the study. (A) SFC numbers detected to ESAT-6 by T-SPOT.TB; (B) SFC numbers detected to CFP-10 by T-SPOT.TB; bars =  medians; (C) IFN-γ responses detected by QFT-GIT in individuals who were initially positive at day 0 compared to day 200 (left) and IFN-γ responses detected by QFT-GIT in individuals who were positive at day 7 compared to day 200. In each case samples from the same individual at these two times are linked by bars. QFT-GIT  =  QuantiFERON Gold in-Tube; IFN-γ  =  interferon-gamma; ESAT-6  =  early secretory antigenic target-6; CFP-10 = culture filtrate protein-10; IU =  international units; SFC =  spot forming cells; Rx =  treatment.

After excluding the presence of active disease, each of the 29 individuals considered positive for LTBI, by either one or both IGRAs at either day 0 or day 7, underwent and completed treatment without complication. Three months after completion the whole cohort provided a third sample. Five of the 27 individuals who provided a positive T-SPOT.TB IGRA at day 0 or day 7 or both times reverted to negativity after they completed treatment (18.5% vs 81.5%, p = 0.22). In those positive by T-SPOT.TB, numbers of ESAT-6 or CFP-10 SFC were not markedly different (Figure 5A and B). Of the 134 negative individuals there was one conversion, though this was weak and in the ‘uncertainty’ zone (data not shown). The agreement between T-SPOT.TB results at day 7 and 200 improved to 96.4% with an ‘optimal’ concordance (κ = 0.86; 95% CI: 0.74–0.97).

Of the 23 individuals positive by QFT-GIT at either day 0 or day 7 or both times, eight reverted to negativity after they had completed their treatment. Median IFN-γ levels decreased by 77.3% following treatment (pre-treatment median IFN-γ 2.73 IU/ml, post-treatment IFN-γ 0.62, p = 0.0002). Of the eight individuals initially positive at day 0, two became negative (median pre-treatment IFN-γ 7.67 IU/ml, post-treatment IFN-γ 2.67 IU/ml, p = 0.063), while of the 15 participants who became positive on day 7 after the TST, 40% reverted (median pre-treatment IFN-γ 1.44 IU/ml, post-treatment IFN-γ 0.49 IU/ml, p = 0.0016) (Figure 5C). Six of the 143 negative participants who did not receive LTBI chemoprophylaxis were positive by QFT-GIT on day 200 (day 7 median IFN-γ 0.02 IU/ml, day 200 median IFN-γ 0.49 IU/ml, p = 0.03), although four of these lay in the zones of uncertainty. Concordance between QFT-GIT results pre- and post-treatment was moderate (89.2%, κ = 0.53; 95% CI:0.34–0.72), and overall agreement between QFT-GIT and T-SPOT.TB results decreased slightly by day 200 to 88.6% (κ = 0.51; 95% CI:0.32–0.70). Therefore, after treatment for LTBI, reductions in IGRA responses, although not consistent, were most detectable in the group positive by QFT-GIT.

## Discussion

This study evaluated the performance of currently available commercial IGRAs for the diagnosis of LTBI in a migrant population, and how antecedent TST and LTBI treatment influence these. The key strengths of this study are the cohort, the similar population age and ethnicity, the assured adherence to treatment, the synchronous manner in which samples were collected and processed and the setting that controlled for TB exposure.

At first presentation nearly three times more individual's had positive TST and T-SPOT.TB results than QFT-GIT results. For all tests there was a greater concordance between an IGRA test and TST in BCG-naive than BCG-vaccinated individuals (Table 1), as previously described [7]. Using a non-stratified threshold to interpret a TST increased the number of positives, but many of these may have been false-positive as half had been BCG-vaccinated. Thus non-stratification may over-estimate the numbers of LTBI individuals, and that in light of results from more specific tests such as IGRAs a stratified threshold may provide a more discriminatory test.

A lack of a ‘gold standard’ test introduces difficulties in evaluating the performance and diagnostic accuracy of assays for detecting LTBI [40]. Comparison with previous studies is also complicated by the heterogeneity in study designs. Nevertheless, systematic reviews and meta-analyses suggest that compared with the TST, IGRAs have a higher specificity for the diagnosis of LTBI and follow up studies show that these tests are better at predicting who will progress to disease [41]. Furthermore, whilst TST specificity is altered after vaccination with BCG, this is not the case for IGRAs and vaccination with BCG [42].

Multiple studies suggest that the T-SPOT.TB assay has a higher sensitivity but somewhat lower specificity than QFT-GIT [7], [8], [42], [43]. A recent observational study of TST and IGRA responses in migrants made the first three-way assessment of screening methods [19]. This identified a ‘fair’ concordance between TST and both IGRAs, and a ‘moderate’ concordance between IGRAs, although simultaneous sampling for all tests was not performed and comparative results were not available for 25% of cases [19]. Clarifying what level of concordance between assays is acceptable and clinically relevant is an important future necessity.

Administration of PPD resulted in a marked increase in QFT-GIT positive individuals, which was not seen for the T-SPOT.TB test. It is unlikely the more numerous T-SPOT.TB positives at day 0 are false positives due to the greater overall diagnostic agreement between TST and T-SPOT.TB results than between TST and QFT-GIT. Additionally, longitudinal, intra-individual T-SPOT.TB results were generally consistent and the TST resulted in QFT-GIT results more reflecting those of the T-SPOT.TB test. Technical factors are unlikely to have influenced QFT-GIT results at day 0 because samples were collected, transported and processed similarly each time. Also, controls were within expected ranges, no indeterminate QFT-GIT results were recorded and the assay was performed by a national TB reference laboratory. We interpret these results to indicate that QFT-GIT has a lower sensitivity for diagnosing LTBI and that tuberculin may enhance the response in some individuals to this test. Further carefully controlled studies are required to confirm these findings.

Results from IGRAs can be dynamic, producing transient responses. Intra-subject variability can complicate their interpretation, particularly for borderline cases, and may occur for reasons including exposure to active TB cases and environmental NTM [11], [25], [44]. In this study, such reasons may be less applicable as participants were confined to a military training facility in a region of England with a low incidence of TB and there was no contact with active disease.

Our data suggest that by assessing IGRA responses before and after TST, five distinct groups can be identified (Table 3). The first two groups are those who are persistently negative or positive, by both IGRAs, throughout the study. The persistently positive results may be due to persisting or higher levels of antigen in LTBI, and occasionally responses increased following further antigenic challenge with PPD. In contrast to others we observed a strong linear correlation between secreted IFN-γ and ELISPOT counts in response to both ESAT-6 and CFP-10 among this group [45].

**Table 3:** Proposed groups classified by IGRA response following TST administration, with suggested possible immune mechanisms.

Group by IGRA response	IGRA assay QFT-GIT/T-SPOT.TB		Supposition
	Pre-TST	Post-TST	
Persistently negative	−/−	−/−	No evidence of previous or current infection
Persistently positive	+/+	++/++	Strong stimulation from persisting antigen accompanied with robust T cell responses to PPD
Concordant conversions	−/−	+/+	Expansion of T cell memory population after PPD stimulation on a background of historic infection
Discordant conversions	−/+	+/++	Expansion of T cell memory population after PPD stimulation on a background of historic infection
Discordant reversions	−/+	−/−	Weak T cell response induced after limited (temporal or quantitative) *M.tb* antigen exposure

IGRA  =  interferon-gamma release assay; TST  =  tuberculin skin test; QFT-GIT  =  QuantiFERON Gold in-Tube; PPD  =  purified protein derivative; +  =  positive response; −  =  negative response.

Among those who converted to positivity during the study we believe there are two groups. The first is comprised of those negative at baseline for both IGRAs and a second group that had discordant IGRA results at baseline. Expansion of memory T cells following PPD stimulation may account for conversion among the first group. The differences in these groups may relate to the level of bacterial burden or antigen availability and therefore the level of immune stimulation. A low sensitivity assay or a weak immune response may produce initial false negative results, which is enhanced after further antigen exposure (i.e. PPD). Differences between the whole-blood ELISA and PBMC ELISPOT may also contribute to the inter-assay variation observed. The final, fifth group of positive to negative reversions only occurred in discordant individuals. One possible explanation is that this may reflect transient exposure to *M.tb* prior to the study, with the results reflecting weak responses declining with time [46]. It is also possible that T cell frequency in peripheral blood is not constant or other reasons such as co-infections modulating test results.

Following completion of treatment, 35% of QFT-GIT-positive individuals reverted to negativity. Median IFN-γ levels decreased significantly in those treated but the change was most profound in those who were positive only after antecedent TST and least marked in those who were positive on day 0. In contrast, responses in the T-SPOT.TB assay were much less affected by chemoprophylaxis. Previous studies have reported variable results for the T cell response to ESAT-6 and CFP-10 in adults following treatment for LTBI for reasons that may include the differing methodologies used and populations examined between studies [28], [35], [36], [47], [48]. Nevertheless, collectively the data suggest that IGRAs alone are sub-optimal tools to monitor the success of treatment for LTBI. Numerous reasons could account for the persistent positivity or modest reductions of IGRA responses following LTBI treatment [48]–[52]. The most critical to understand is whether treatment actually clears LTBI or if it simply enables better control of the latent infection. There may also be differences in the rate of decline of effector T cell numbers or their production of IFN-γ, possibly reflecting antigen persisting after bacillary death. None of these reasons are mutually exclusive. Future studies are needed to understand the kinetics of immunological responses in individuals with LTBI and the levels of sterilization achieved by chemoprophylactic treatment.

There are a number of limitations to this study. First is the lack of a true ‘gold standard’ for diagnosing LTBI which is an issue shared with other studies in this field. Furthermore, there was no untreated LTBI control group and also no regular, longitudinal follow-up of individual patients. Additionally, although unlikely to have any persisting effect, antecedent TST administration may have confounded the response of an individual to treatment.

In summary, this study has examined the response in two commercially available IGRAs for the diagnosis of LTBI in a migrant population of Gurkha recruits. The study finds that in the absence of a TST then more positive responses could be identified by the T-SPOT.TB test than by QFT-GIT. Nevertheless, after a recent TST the responsiveness to both tests was more similar, mainly due to an increase in the numbers of positive QFT-GIT responses. Follow-up IGRA testing for LTBI after treatment in this population supports the current concept that monitoring of IGRA responses in adults following completion of chemoprophylaxis is of speculative value only.

In light of the findings presented in this study the UK Ministry of Defence has altered its policy for LTBI screening in Gurkha recruits to the use of a single T-SPOT.TB IGRA [53]. These findings are important because they have implications for LTBI diagnostic strategies in populations of migrants arriving in countries of low TB incidence from areas of high TB incidence. This may also have some cost and time saving implications for screening in non-military migrants in the U.K. and thus may help to inform future UK guidance.
